# Oral health education strategies for patients living with cardiovascular disease within hospital settings: a scoping review

**DOI:** 10.3389/fpubh.2024.1389853

**Published:** 2024-06-19

**Authors:** L. A. Church, L. Robins, F. Xu, L. Qin, A. Tran, J. P. Wallace, S. King

**Affiliations:** ^1^The University of Sydney Dental School, The University of Sydney, Sydney, NSW, Australia; ^2^Westmead Applied Research Centre, The University of Sydney, Westmead, NSW, Australia; ^3^School of Health Sciences, Oral Health, The University of Newcastle, Ourimbah, NSW, Australia

**Keywords:** oral health, cardiovascular disease, health promotion, oral health education, prevention

## Abstract

**Objective:**

To identify and describe the impact of current oral health education programmes provided to patients in cardiology hospital wards and outpatient clinics.

**Methods:**

This review was conducted in accordance with the Preferred Reporting Items for Systematic Reviews and Meta-Analysis for Scoping Reviews statement. Searches were conducted using electronic databases: Cochrane, Medline, and Scopus, as well as grey literature searching.

**Results:**

Three eligible studies were identified. All included studies reported generalised poor oral health in their participants at baseline, with significant improvement at follow-up. They all reported significant reductions in plaque deposits and gingival bleeding. One study reported significantly less bacteria on participant tongues, as well as fewer days with post-operative atrial fibrillation in the intervention group. Furthermore, in this study, one patient in the intervention group developed pneumonia, whilst four patients in the control group did.

**Conclusion:**

Oral health education for patients with cardiovascular disease is limited and many have poor oral health. Educational programmes to improve oral health behaviours in patients with cardiovascular disease can improve both oral and general health outcomes.

**Implications for public health:**

Oral disease is a modifiable risk factor for cardiovascular disease. Integrating oral health education into cardiology hospital settings is a simple strategy to improve access to oral health information and improve both oral and cardiovascular outcomes.

## 1 Introduction

Cardiovascular disease (CVD) is a global public health issue. It is the leading cause of death world-wide (1) and whilst modifiable risk factors smoking status, healthy diet, active lifestyle, and alcohol intake are well known (2, 3); one that is rarely publicised is poor oral health. Up to 90% of any population with at least one tooth are living with periodontal disease, a preventable oral condition that plays an integral role in oral as well as systemic health. Preventing this and other oral diseases begins with oral health education and involves oral hygiene instructions.

Optimal oral hygiene practices involve toothbrushing for at least 2 mins twice daily (4, 5) and cleaning interdentally once a day (6). Manually removing dental biofilm from oral tissues with these habits are the most efficient way to ensure good oral health (7) and lower both local and systemic inflammation (8). Traditionally oral health education is delivered within dental settings. However, just under half of adults world-wide do not attend a dentist regularly (9) and as such, do not receive this important messaging.

The health outcomes of those living with CVD can be impacted by poor oral hygiene as never or rarely brushing teeth has been shown to significantly increase the risk of a CVD event (10). The mechanism behind this is a result of poor oral hygiene allowing dental biofilm to remain stagnant on oral tissues, initiating an immune response and involves vasodilation of gingival tissues to allow rapid movement of immune cells to the site (11). As such, even in healthy individuals, poor oral hygiene can lead to elevation of inflammatory markers high-sensitive C-reactive protein (hsCRP) and interleukin (IL)-6 in as little as 3 weeks (12). For those living with CVD, an elevation of these markers puts them at an increased risk of a future cardiac event (13).

Vasodilation of gingival tissues also gives pathogenic oral bacteria within the dental biofilm access to the body and its systems via the blood stream (14–16). Once in the bloodstream these pathogens and their secretions can lodge in distant organs such as the lungs, kidneys, brain (14), and heart (17) where they can initiate a localised inflammatory response (18). In the heart these pathogens have been shown to invade vessel walls and adhere to atherosclerotic lesions, leading to atherosclerosis (19); the primary cause of CVD (20).

Many barriers exist that prevent individuals from attending the dental clinic such as cost, ease of access (21), and anxiety (22) to name a few, highlighting the need to expand oral health education to other areas of healthcare. Research has shown that increased oral hygiene in hospital wards decreases the incidence of non-ventilator hospital acquired pneumonia (23) and shortens hospital stays (24). Within this setting, however, oral care is delegated to nurses who can face many challenges to providing this care (25). As such, oral health practitioners (OHP) would be an appropriate alternative to take on this responsibility (26).

Equally, an emerging educational tool within healthcare has been the use of digital devices (27). Their ability to deliver health messages across all literacy levels (28, 29) has improved patient quality of life (QoL) (30, 31); and digitally delivered health information within patient waiting rooms has been shown to improve oral hygiene practices (32) and promote healthy lifestyle behaviours in patients with CVD (33). Whether face-to-face or digitally delivered, oral health education provided in cardiology settings has the potential to improve oral health and reduce the risk profile of patients living with CVD.

### 1.1 Aims

This review aims to identify and describe oral health education programmes provided to patients living with CVD within hospital wards and outpatient clinics; as well as discuss any effect they had on health outcomes.

## 2 Methods

This review was conducted in accordance with the Preferred Reporting Items for Systematic Reviews and Meta-Analysis for Scoping Reviews (PRISMA-ScR) statement (34). See Supplementary material 1. The review protocol is registered in the Open Science Framework (OSF) registry.^1^

### 2.1 Search strategy

The initial search commenced 21st December 2022 and was repeated on 14th August 2023. The final search occurring 7th May 2024. Electronic databases used include Cochrane, Medline (via Ovid), and Scopus. No limitations were placed on language or publication period, and no human filter was applied. Grey literature included phrase searching via Google Scholar, as well as reviewing citation lists of relevant studies. The search strategy included a combination of the following terms *“cardiovascular disease, heart disease, education relating to dental health, oral health, health promotion, digital education, video education, patient education, health knowledge, oral hygiene instruction, hospital, oral health, oral care, dental care, preventative dentistry, video recording, video-audio media.”* Boolean operators (AND/OR), medical subject headings (MeSH), and truncations were also utilised. For the full search see Supplementary material 2. Every attempt was made to retrieve studies that were inaccessible including web searches, using The University of Sydney library resources, and lastly, attempting to contact corresponding authors.

### 2.2 Inclusion and exclusion criteria

The population comprised of adults ≥18 years who had been or were recently hospitalised as a result of cardiovascular disease. The intervention included digital and/or traditional oral health education being delivered to these patients within hospital wards or out-patient clinics. Any studies where oral hygiene education took place outside of the hospital environment, or within a dental setting were excluded. Only peer-reviewed publications of randomised controlled trials, quasi-randomised controlled trials, observational studies, including cohort, case–control and cross-sectional studies were eligible for inclusion. Conference abstracts, case-studies/series, letters to the editors, and editorials were excluded. Any non-English or animal studies were manually excluded.

### 2.3 Screening and data extraction

**Figure 1:**
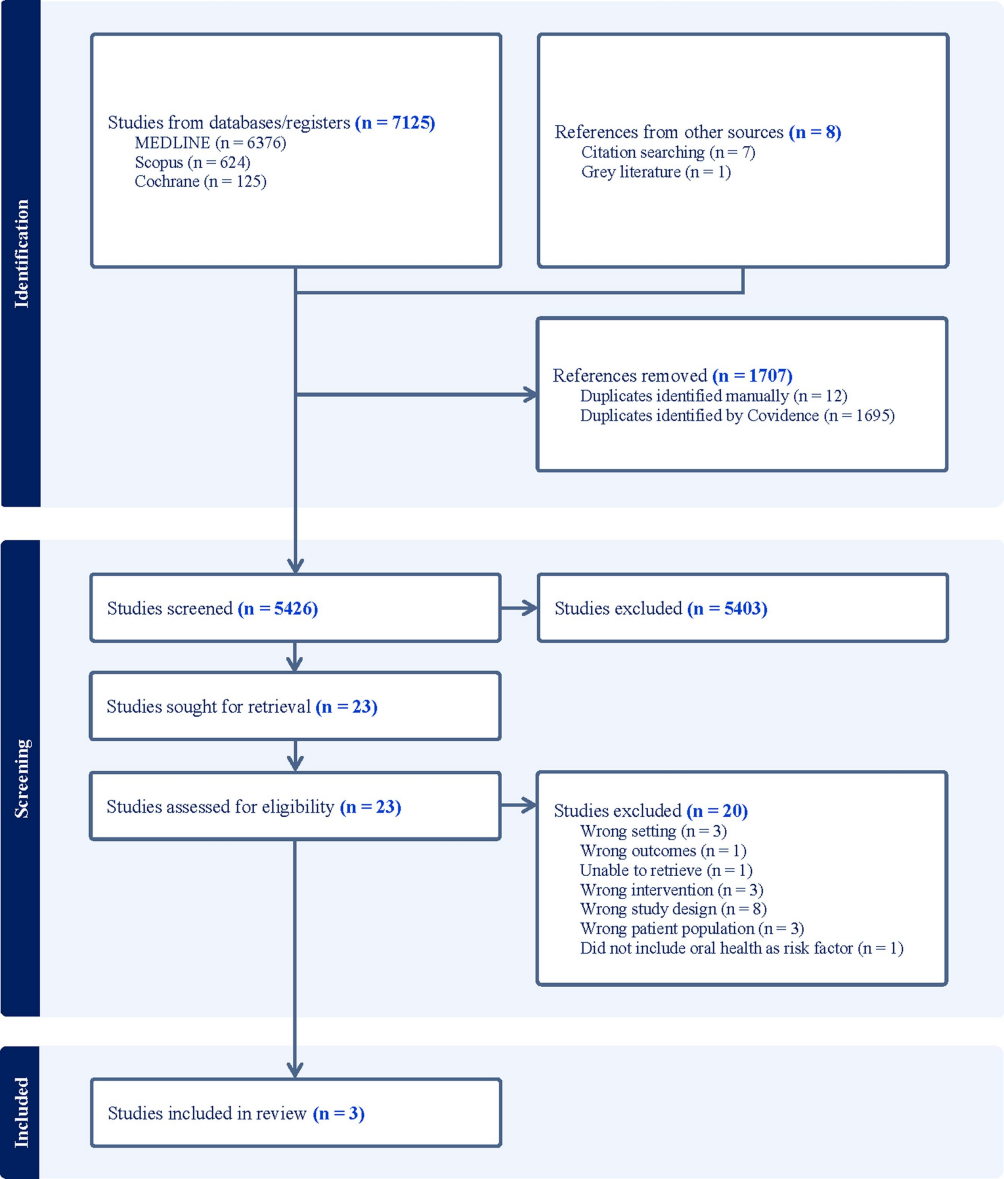
Study identification PRISMA flow diagram.

Using Covidence, a systematic review software (35), 7,133 identified citations were imported into the programme where 1,695 duplicates were automatically removed. A further 12 duplicates were manually removed leaving a total of 5,426 for screening. Titles and abstracts were screened independently by LC and one of four alternate reviewers (LR, AT, LQ, FX). Most results did not relate to oral health education, CVD, adults, and/or were not based within a hospital setting. As such they were deemed irrelevant. Twenty-three articles were identified for further assessment, however after full text screening, a total of 3 studies [1 quasi-randomised (36) and 2 randomised controlled trials (37, 38)] met the inclusion criteria (see Figure 1). Any conflicts arising during the screening process were resolved via group discussion. A data extraction tool developed within Covidence was completed independently first by LC, followed by either LR, FX, AT, or LQ. Key characteristics extracted were author(s), publication year, country, study design, aim(s), setting, mean age, sex, outcomes measures, a comparison of oral hygiene intervention, intervention duration, and outcomes. Due to the heterogeneity of the studies, data synthesis has been presented narratively with reference to supporting material.

## 3 Results

### 3.1 Characteristics

Three studies were identified (36–38), and included a total of 245 participants. See Table 1. The studies took place in either Hong Kong or Japan and spanned between 2013 and 2019. Participants recruited into the studies were patients who were within either a cardiac surgical or stroke rehabilitation hospital ward or were attending a hospital out-patient rehabilitation clinic. Across all studies, the lowest mean age of participants was 66.6 ± 10.8, the highest 70.9 ± 11.1; 60.6–68% were male. Whilst Omori et al. (36) did not discuss employment status, two thirds of Dai et al.’s (37) (housewife: 12.8%, retired: 51.1%, and unemployed: 2.1%), and close to three quarters of Lam et al.’s (38) subjects were not working (53.1% retired, 19.8% homemaker). All studies reported a lack of regular oral hygiene practices at baseline (36–38), and reported no significant difference in oral hygiene status between intervention groups at baseline.

**Table 1:** Included study characteristics.

Authors Year	Country	Design	Aim	Setting	Total participants	Age (Mean ± SD)	Sex (M/F)	Outcome measure
Lam et al. (38) 2013	Hong Kong	RCT	To evaluate the effectiveness of oral health promotion interventions on clinical oral health among patients after stroke in acute and rehabilitative hospital settings.	In-hospital stroke rehabilitation ward.	80	66.9 ± 10.9	51/30	Silness and Löe Plaque Index (39, 40) Gingival Bleeding Index (41) Oral functional status
Dai et al. (37) 2017	Hong Kong	RCT	To compare the effectiveness of an advanced oral hygiene care programme to a conventional oral hygiene in controlling plaque levels and reducing gingival bleeding among patients with stroke who had normal cognitive abilities during outpatient rehabilitation.	Out-patient stroke rehabilitation clinic within Tung Wah Hospital, Hong Kong	94	66.6 ± 10.8	57/37	Silness and Löe Plaque Index (39, 40) Gingival Bleeding index (41)
Omori et al. (36) 2019	Japan	Quasi-RCT	To determine the effects of six-step method on postoperative numbers of oral bacteria, periodontal status, and atrial fibrillation among inpatients with heart diseases and periodontitis.	Department of Cardiovascular Surgery, Kagawa University Hospital	70	Control: 70.9 ± 11.1 Intervention: 68.1 ± 11.4	48/22	Primary: Number of oral bacteria on the tongue. Secondary: Oral hygiene status via Number of teeth and DMFT PCR%, BOP%, Periodontal parameters via mean PPD(mm) and PPD ≥4 mm (%). Tongue coating scores, Self-efficacy scale for self-care (SESS), Incidence of postoperative AF.

### 3.2 Outcome measures

The primary outcome measures for two studies (37, 38) included measures of oral hygiene status using the Silness and Löe plaque index (39, 40) and the gingival bleeding index (41). See Tables 2, 3 for indices criteria. Secondary outcomes included gingival bleeding at 6 months (37) or oral functional status, assessed by patients’ ability to perform toothbrushing and insert/remove their dentures (38). The outcome measures for one study (36) was the number of oral bacteria on the tongue, followed by oral hygiene status, periodontal parameters, tongue coating scores, self-efficacy scale for self-care (SESS) scores, and the incidence of postoperative atrial fibrillation (AF). This study also assessed plaque score by using O’Leary’s plaque control record method (43).

**Table 2:** Criteria for Silness and Löe plaque index and gingival bleeding index (42).

Code	Plaque index criteria
0	No plaque.
1	A film of plaque adhering to the free gingival margin and adjacent area of the tooth. The plaque may be recognised only after application of disclosing agent or by running the explorer across the tooth surface.
2	Moderate accumulation of soft deposits within the gingival pocket that can be seen with the naked eye or on the tooth and gingival margin.
3	Abundance of soft matter within the gingival pocket and/or on the tooth and gingival margin.

**Table 3:** Criteria for gingival bleeding index (36–38).

Code	Gingival bleeding index criteria
0	No bleeding
1	Bleeding

### 3.3 Interventions

The education provided in each trial focused on oral hygiene instruction and was delivered by oral health practitioners or dental assistants. Each intervention differed in its methods and materials, with follow ups of 3-weeks (38), 3- and 6-months (37), and discharge (approximately 1 month) (36). See Table 4 for intervention details. In the first study, stroke patients attending their outpatient rehabilitation were placed into one of two arms control: conventional oral hygiene care programme (COHCP) or intervention advanced oral hygiene care programme (AOHCP). The control arm receiving an oral hygiene care programme, manual toothbrush, regular toothpaste (Colgate), and one on one oral hygiene instruction with a dental assistant. Whilst the intervention arm received the care programme, toothpaste, and one on one instruction however, also received an electric toothbrush with manufacturer’s instructions and a 3-month supply of chlorhexidine (CHX) mouth rinse (37).

**Table 4:** Oral hygiene intervention and findings.

Authors Year	Intervention duration	Control	Intervention 1	Intervention 2	Findings
Lam et al. (38) 2013	3 weeks	Oral Hygiene Instruction provided by a registered dentist.	Oral Hygiene Instruction provided by a registered dentist. 2 x daily Chlorhexidine mouth rinse use	Oral Hygiene Instruction provided by a registered dentist. 2 x daily Chlorhexidine mouth rinse use 2 x weekly assisted brushing	Statistically significant reductions (*p* = <0.001) in PI scores were observed in all groups over the clinical trial period. Reductions in plaque index scores were significantly greater in the two intervention groups receiving CHX. Significant reductions in median gingival bleeding index scores were observed only in intervention groups (*p* = 0.003). No patients developed pneumonia over the course of the clinical trial, however authors admit this absence may have been a result of the small sample size. Left side hemiplegia experienced significantly smaller reductions in mean PI scores compared with patients with right side hemiplegia. At the time of baseline assessment 67.9% of participants reported not to have a regular daily brushing habit (at least once daily). By the end of the RCT, 91.4% of the sample had adopted a regular brushing habit.
Dai et al. (37) 2017	3- & 6-months	Conventional oral hygiene care programme Supply of a manual toothbrush Regular toothpaste (Colgate) Oral hygiene training, one on one with a dental surgery assistant	Conventional oral hygiene care programme Supply of a powered toothbrush Regular toothpaste (Colgate) Oral hygiene training, one on one with a dental surgery assistant Manufacturers instructions for the powered toothbrush 3-month supply of 0.2% chlorhexidine mouth rinse	N/A	The study was effective in reducing individual plaque index and gingival bleeding. There was a significant reduction in the percentage of sites with moderate to abundant plaque over time within each group: AOHCP (powered toothbrush): *p* = <0.001 and COHCP (manual toothbrush): *p* = <0.001. There was a significant reduction in the percentage of sites with gingival bleeding over time within each group (AOHCP: *p* < 0.001 and COHCP: *p* < 0.05). The AOHCP group experienced a higher incidence of staining than the COHCP group, which was attributed to the use of the chlorhexidine mouth rinse. 73.4% of participants reported not to perform regular toothbrushing daily, showing evidence of inadequate oral hygiene care and thus the importance of considering incorporating oral hygiene care in stroke rehabilitation needs to be advocated.
Omori et al. (36) 2019	Two weeks after surgery, and/or discharge.	Teaching oral hygiene skills for 15 min per week Disclosing dental plaque accumulation Teaching how to use interdental brushes	Teaching oral hygiene skills using the six-step counselling method for 15 min per week Disclosing dental plaque accumulation Teaching how to use interdental brushes.	N/A	Two weeks after surgery, or at discharge, there were significant decreases in the intervention group compared to the control for all study outcomes including: Fewer oral bacteria found on the tongue (*p* = < 0.001; effect size was large). Decreases in the amount of tongue coating and plaque control record scores (*p* = <0.001; effect size was large). Decreases in mean PPD (mm), PPD ≥4 mm (%), and BOP (%) (*p* < 0.05; effect size was large except for mean PPD). Change in self-efficacy score at discharge (*p* = 0.001; effect size was large), Self-efficacy score worsened in the control group. After adjustment, postoperative AF (day) persisted for less time after adjustment: 4.8 ± 7.6 vs. 1.5 ± 2.5 days, respectively, (*p* = 0.019; effect size was medium). Oral health behaviour [frequency of toothbrushing (≥ 2/day) and interdental brushing] at discharge was improved compared to the baseline in both groups. At discharge the number of patients who reported the use of interdental brushing in the intervention group was significantly larger than that in the control group. [33 (94.3%) vs. 19 (54.3%)] (*p* < 0.001). While not reaching significant levels, the incidence of postoperative pneumonia was lower in the intervention, than the control group (2.9% vs. 11.4%).

Similarly, investigators from another study involving CVD patients within a surgical ward (36), placed them into one of two arms. Both arms received similar interventions including oral hygiene instruction, using disclosing solution, interdental brush use delivered by certified dental hygienists. The hygienists also provided post-operative oral care to a small number of participants in each group. However, the teaching method differed between the groups. The control group received skills-based teaching, the intervention arm received oral hygiene instruction via a modified behavioural six-step method (44). See Table 5 for this method.

**Table 5:** Six-step method adapted from included study (36).

Step	Modification
1: Identification of the problem	Knowledge, belief, and barriers to self-care were clarified by a dental hygienist in interviews as well as using disclosing solution so each patient could determine areas that they were not brushing appropriately. Thus identifying their problems.
2: Creation of commitment and confidence	The clinical interview and counselling were expanded. The patients learned the importance of maintaining good perioperative oral hygiene status. A dental hygienist encouraged the patients to confirm their intention and to promote motivation.
3: Increase awareness of behaviour	Patient awareness of their own behaviour was increased through self-monitoring. A dental hygienist instructed the patients to maintain a diary regarding toothbrushing and the use of interdental brushes to monitor their accomplishments and identify barriers to changes in behaviour.
4: Development and implementation of an action plan	Short-term action plans established according to the principle of gradualism were based on the skill, behaviour, and oral hygiene status of each patient. These plans were concrete, realistic, achievable, and included “brush teeth three times each day,” “use interdental brushes daily,” or “clean the tongue every day.” Patients set goals that they could achieve by the time of the next interview.
5: Evaluation of the plan	Whether or not the patient implemented the action plan was evaluated. Successful experiences were acknowledged and supported. When the plan succeeded, the success experience was acknowledged. The dental hygienist praised improvements in the oral hygiene status of the patients, even if quite small. Failure was attributed to failure of the plan, and a new achievable plan was established.
6: Maintenance of change and prevention of relapse	Some inpatients had perioperative high-risk situations that resulted in relapse; for instance, postoperative poor physical status, a sink located far from their hospital bed, or limited range of hand movement due to intravenous drips. Thus, it was important for the dental hygienist to safeguard and reinforce the new behaviours to help and encourage the patients.

The final study (38) included three arms: one control and two interventions. Each arm received oral hygiene instruction, whilst the intervention arms also received chlorhexidine (CHX) mouth rinse alone or in combination with 2 x weekly assisted brushing. The hygiene instruction was performed by a registered dentist and CHX was administered by ward nurses. The intervention arm receiving assisted brushing, had this performed by trained ward nurses. Training involved a 30-min education session run by dental hygienists. The authors deemed it unethical to include a negative control group due to their high risk of developing aspirational pneumonia (45).

### 3.4 Findings

All 3 studies reported a lack of regular oral hygiene practices at baseline (36–38). At their conclusion, all found an improvement in toothbrushing habits, whilst one (36) reported a significant increase of interdental brush use. All study arms had significant reductions in oral hygiene measures including plaque scores (*p* = <0.001) (36–38), improved periodontal parameters, and tongue coating scores (36). Gingival bleeding was also reduced in all arms of two studies (p 0.004) (36), (*p* < 0.001) (37), however, one (38) reported significance in the intervention groups only (*p* = 0.032). One study assessed tongue bacterial numbers (36), and reported significantly less bacteria (x10^7^cfu/mL) on participant tongues (*p* < 0.02), as well as fewer days with post-operative AF in the intervention group (1.5 ± 2.8 vs. 4.8 ± 7.6 *p* = 0.019). It also reported that 5 patients (4 control and 1 intervention) developed pneumonia (36), whereas no patients developed pneumonia in the other studies (37, 38). A 6-month follow-up was conducted in one study and they reported the continuation of plaque reduction (*p* = 0.05) and a further reduction in bleeding on probing (*p* < 0.01) in their intervention arm (37). SESS was measured in one study, showing worsened scores in the control arm (36).

## 4 Discussion

This review assessed the current evidence in relation to oral hygiene education programmes provided to patients within cardiology wards and/or outpatient clinics and found that oral health education is rarely provided in these settings. Poor oral health is a significant global public health issue (46). Essential to preventing poor oral health is oral health education. However, as patients can have major barriers to overcome when accessing dental care (47), oral health education urgently needs to expand beyond the dental clinic. Incorporating this education and improving oral health within other areas of health would greatly benefit everyone and would have a profound positive effect on patients living with CVD (3–6).

At baseline, participants of all included studies had poor oral health as well as suboptimal hygiene habits (36–38). At the end of the study periods, the intervention groups saw significant improvement in both clinical oral health status and self-reported oral hygiene habits. These findings mirror other studies using oral health practitioners (OHP) to educate patients in non-dental settings including residential aged care (48, 49) and mental health facilities (50–53).

Omori et al. (36) specifically illustrated a significant increase of interdental brush use in their study [(94.3%) intervention vs. (54.3%) control]. The improvement of interdental brush use, as well as other outcome parameters within the intervention arm is likely due to the six-step teaching method. This method has been shown to improve health outcomes as clinicians collaborate with patients to set achievable goals (44). Skills-only based health education is more paternalistic in nature, excluding patients’ prior beliefs or understanding, and removing autonomy (54). As the control arm received this type skills-based education, it could be related to the worsening of SESS score in this group.

The improvement of oral health reported in the included studies also had a positive impact on post-operative health outcomes (36–38) such as a reduction or absence of common CVD post-operative complications: pneumonia and post-operative AF; which can lengthen hospital stays or cause premature death (55–57). Although good oral hygiene has been shown to reduce systemic inflammation (58) none of the included studies reported on inflammatory markers such as hsCRP or IL-6.

### 4.1 Current education strategies

The education strategies employed in all included studies involved traditional face-to-face education (36–38) and many of the excluded studies provided this education to nursing staff only (59–63). Nurses can face many challenges when providing oral care. From a personal level, barriers can include staffing issues, lack of time or training, or aversion to this care (25). This is reflected by a study where nurses admitted to ceasing toothbrushing altogether after the study period, even after oral hygiene was proven to eliminate ventilator assisted pneumonia due to the lack of time and because oral care was of low priority (61). However, challenges can also arise at the organisational level where training, resources and/or appropriate staff numbers are not provided (25).

Furthermore, patients themselves can prevent nurses from providing oral care with aggressive behaviour, care refusal, communication issues, or where oral health is not prioritised (25). As such OHPs including dentists, dental hygienists, and oral health therapists, could be an appropriate alternative to help ease this burden on nursing staff (26). The FDI World Dental Federation also recognises the benefit OHPs would have within primary healthcare settings, calling for them to be integrated into these settings globally by 2030 (64). Currently however, resulting from a lack of political leadership, low oral health prioritisation on political agendas, as well as staffing, infrastructure, and funding obstacles (65), few countries are taking steps to utilise them in this way (66).

### 4.2 Changing the status quo

One cost-effective way to bring oral health education to patients in these settings is through the use of digital technology. Digital CVD education programmes have been used to improve heart health outcomes (30, 33), including text-message health tips post cardiac event (67). Whilst currently oral health messages are not included, they could easily be incorporated into these existing education packages. Another solution could be providing oral health training to non-dental clinicians such as pharmacists, general practitioners, and other allied health professionals, as many individuals visit these clinicians when they have a dental issue (68).

A long-term solution at the organisational level could be a collaboration between universities and hospitals forming placements for student OHPs. Placements such as this have shown to benefit both students, hospital staff, and patients alike (69). Students placed in hospital settings would provide oral health education, assist with oral care, and with mobile dental units becoming more readily available, urgent treatment could be completed bedside (70). Additionally, referral pathways within the hospital system could also be created, utilising hospital dental clinics where applicable.

### 4.3 Gaps in the literature

The number of eligible studies that involved the direct provision of oral health education to patients with CVD within hospital settings were limited and predominately located in Hong Kong (37, 38) or Japan (36) between 2013 and 2019. These findings highlight the need to conduct more studies in different global communities. All included studies reported generalised poor oral health in their participants at baseline, similar to recent research within a Romanian emergency hospital (71) and an Australian cardiac rehabilitation clinic (72). Both concluding further oral health education in these spaces are needed (71, 72).

A lack of resources and funding means OHPs are absent from hospital multidisciplinary teams (57, 58). Poor oral health is a modifiable risk factor for CVD however, Appropriately, priority is given to patients’ heart health in cardiology wards however, current literature acknowledges poor awareness of the links between oral and heart health in patients within cardiology wards and outpatient clinics (58, 59). This could be related to the absence of an OHP within these settings and compounded by the limited oral health messages in CVD education (25, 60–62).

This review has discussed oral health education programmes provided to patients with CVD in hospital settings. It has highlighted gaps where an OHP and/or digital technologies would be ideally placed to bridge them. As such, there are opportunities for future research and implementation of oral health education programmes for patients with CVD within hospital settings. Preferably oral health education should form part of primary prevention strategies for good general health, however incorporating it as part of secondary prevention strategies should also be a priority.

### 4.4 Clinical significance

This review has highlighted the significant role oral health education plays in improving the long-term oral health in patients within hospital settings, as well as lowering the risk of common post-operative and post-stroke complications. Despite the important role oral health can play in cardiovascular health, this review has highlighted a lack of oral health education available to patients with CVD and proposed simple strategies to deliver these messages. The implementation and standardisation of programmes such as these may help to empower at-risk patients at their most vulnerable to improve their oral health for better general health.

### 4.5 Strengths and limitations

The small number of eligible studies was a major limitation for this review. The three included studies took place in Hong Kong or Japan and thus may not be generalisable to CVD patients globally. However, a strength of this study is it is the first known review to analyse oral health education programmes provided to patients with CVD in hospital settings, highlighting a lack of oral health education in these spaces.

## 5 Conclusion

This review concludes there is a need for further development and evaluation of oral health education programmes within hospital settings in different countries. Many patients with CVD have poor oral hygiene which can increase their risk of a recurrent cardiac event. The provision of basic oral health education provided directly to patients significantly improved oral hygiene, minimised the risk of post-operative pneumonia, and lowered post-operative days with AF.

## Data availability statement

The original contributions presented in the study are included in the article/Supplementary material, further inquiries can be directed to the corresponding author.

## Author contributions

LC: Conceptualization, Data curation, Formal analysis, Investigation, Methodology, Writing – original draft, Writing – review & editing. LR: Formal analysis, Writing – review & editing. FX: Formal analysis, Writing – review & editing. LQ: Formal analysis, Writing – review & editing. AT: Formal analysis, Writing – review & editing. JW: Conceptualization, Writing – review & editing. SK: Supervision, Writing – review & editing.

## Footnotes

^1^ https://doi.org/10.17605/OSF.IO/V3HPW
