# Supplementary material for: Oral health education strategies for patients living with cardiovascular disease within hospital settings: a scoping review
Source: Front Public Health. 2024 Jun 19;12:1389853. doi: 10.3389/fpubh.2024.1389853 (PMC11220159; doi:10.3389/fpubh.2024.1389853)
Supplement: Supplementary file 1 [file Table_1.DOCX]

| **Database** | **Search terms** |
| --- | --- |
| **Medline** | cardiovascular.mp. or Heart/ or Heart Diseases/ and Dentistry/ed [Education] or Health Promotion/ed [Education] or Oral Health/ed [Education] or Preventive Dentistry/ or Oral Health/ or Dental Care/ or Dentistry/ or behaviour.mp. |
|  | Dentistry/ed [Education] or Health Promotion/ed [Education] or Oral Health/ed [Education] or Preventive Dentistry/ or Oral Health/ or Dental Care/ or Dentistry/ and cardiovascular.mp. or Heart/ or Heart Diseases. |
|  | Dentistry/ed [Education] or Health Promotion/ed [Education] or Oral Health/ed [Education] or Preventive Dentistry/ or Oral Health/ or Dental Care/ or Dentistry/ and cardiovascular.mp. or Heart/ or Heart Diseases and educational videos.mp. or Health Knowledge, Attitudes, Practice/ or Patient Education |
|  | cardiovascular.mp OR Heart/ or Heart Diseases/ AND Dentistry/ed [Education] OR Health Promotion/ed [Education] OR Oral Health/ed [Education] OR Preventive Dentistry/ or Oral Health/ or Dental Care/ or Dentistry/ AND educational videos.mp. or Health Knowledge, Attitudes, Practice/ or Patient Education as Topic/ or video.mp. or Video Recording/ or Video-Audio Media/; |
| **Cochrane** | ("cardiovascular disease" OR "heart disease"):ti,ab,kw AND ("oral health education" OR "dental health education" OR "oral hygiene instruction"):ti,ab,kw (Word variations have been searched) |
|  | ("cardiovascular disease" OR "heart disease"):ti,ab,kw AND ("oral health education" OR "dental health education" OR "oral hygiene instruction"):ti,ab,kw AND (hospital*):ti,ab,kw |
|  | ("cardiovascular disease" OR "heart disease"):ti,ab,kw AND ("oral health education" OR "dental health education" OR "oral hygiene instruction"):ti,ab,kw AND (education):ti,ab,kw |
|  | ("cardiovascular disease" OR "heart disease"):ti,ab,kw AND ("oral health education" OR "dental health education" OR "oral hygiene instruction"):ti,ab,kw AND ("digital" OR "video") |
| **Scopus** | "cardiovascular disease" OR "heart disease" AND "oral health education" OR "dental health education" OR "oral hygiene instruction" AND "digital education" OR "video education" |
|  | "cardiovascular disease" OR "heart disease" AND "oral health education" OR "dental health education" OR "oral hygiene instruction" |
| **Grey searching** | "oral hygiene instruction in hospital wards" "oral health promotion in a non-dental setting" "oral health promotion in hospital" "oral hygiene instructions in hospital" "oral hygiene instructions in cardiology ward" |

**Supplementary material 1.** Database search strategies.
